# The Crucial Role of Bedside Echocardiography and Clinical Suspicion in Diagnosing Pulmonary Embolism With ST Elevation on ECG, Initially Misdiagnosed as ST-Segment Elevation Myocardial Infarction (STEMI)

**DOI:** 10.7759/cureus.76417

**Published:** 2024-12-26

**Authors:** Mohamed Ghonem, Islam Abdelraouf, Wilsonne Chua

**Affiliations:** 1 Cardiology, Glenfield Hospital, University Hospitals of Leicester NHS Trust, Leicester, GBR; 2 Cardiology, University Hospitals of Leicester NHS Trust, Leicester, GBR; 3 Medicine, University Hospitals of Leicester NHS Trust, Leicester, GBR

**Keywords:** clinical suspicion, echocardiography, pulmonary embolism, right ventricular strain, stemi

## Abstract

Pulmonary embolism (PE) is a life-threatening condition with varied presentations, occasionally mimicking ST-segment elevation myocardial infarction (STEMI). This case highlights a 52-year-old male patient with a history of venous thromboembolism (VTE) who presented with progressive shortness of breath over a month, culminating in dyspnea at rest, and anterior ST-segment elevation on electrocardiography (ECG). The initial evaluation suggested STEMI. Notably, chest pain, a typical feature of STEMI, was absent. This combined with the patient’s clinical background and shortness of breath as presenting symptoms prompted further investigation. Bedside echocardiography revealed right ventricular dilation and dysfunction, and computed tomography (CT) pulmonary angiography confirmed massive PE. Despite anticoagulation and mechanical thrombectomy, the patient succumbed to complications before pulmonary endarterectomy. This report underscores the importance of integrating clinical acumen, advanced imaging modalities, and timely multidisciplinary collaboration to avoid misdiagnosis and optimize patient outcomes in critical cases.

## Introduction

Pulmonary embolism (PE) can occasionally present with electrocardiographic findings that mimic ST-segment elevation myocardial infarction (STEMI), such as ST elevations in anterior leads. This rare presentation poses a diagnostic challenge, as it closely resembles acute coronary syndrome, often prompting emergency cardiac catheterization to rule out coronary artery occlusion [1]. The ST elevation in PE is primarily attributed to acute right ventricular (RV) strain or ischemia resulting from elevated RV pressure due to an acute increase in pulmonary vascular resistance (PVR). Acute RV strain leads to electrical disturbances in the heart, including rightward shifts in the QRS axis and repolarization abnormalities, which may manifest as ST-segment elevations in anterior leads [2]. Diagnostic differentiation between PE and STEMI in emergency settings is critical. While bedside echocardiography can reveal RV dilation and dysfunction (e.g., McConnell’s sign), CT pulmonary angiography remains the gold standard for confirming PE. Additionally, atypical ST-segment patterns, such as a lack of reciprocal changes or dynamic evolution typically seen in STEMI, can serve as clues for PE [3]. The Pulmonary Embolism Severity Index (PESI) and the simplified Pulmonary Embolism Severity Index (sPESI) are the most commonly used scores to predict 30-day mortality in patients with an established diagnosis of PE signifying the importance of early diagnosis and management of PE [4].

## Case presentation

**Figure 1:**
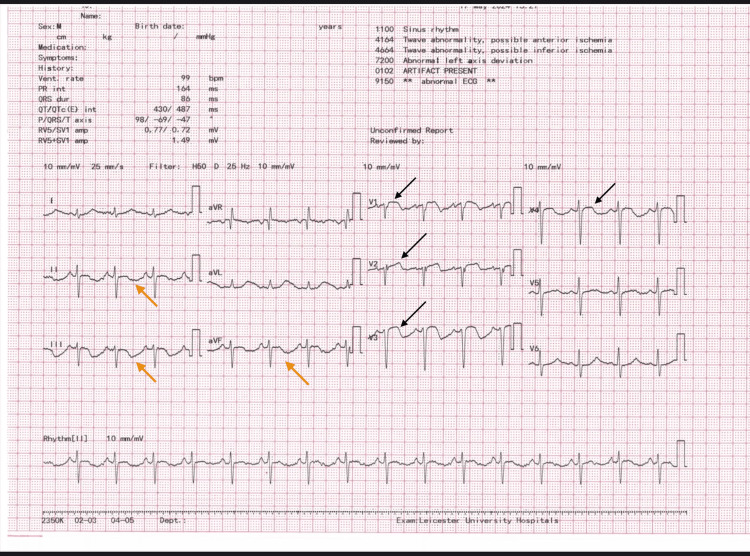
ECG showing ST-elevation V1-V4 (black arrows) with T wave inversion in inferior leads (orange arrows).

A 52-year-old male patient who was an ex-smoker, with a past medical history of hypertension and previous episodes of deep vein thrombosis and pulmonary embolism, treated with anticoagulation four years prior was initially diagnosed by the triage team to have acute ST-elevation myocardial infarction and urgently referred to cardiology for further management. The patient presented with worsening shortness of breath over one month, with a progressive course now occurring even at rest associated with chest tightness. Vital signs were stable, systemic examination was unremarkable, and electrocardiography (ECG) showed ST-segment elevation in leads V1-V4 and deep T-wave inversions in the inferior leads (Figure 1).

While blood test results were initially pending, the patient’s primary presenting symptom was shortness of breath, without clear chest pain. Given the past medical history suggestive of PE and the atypical nature of the ST-segment changes for STEMI, suspicion of PE was raised. The best available bedside diagnostic tool to differentiate and support this suspicion was an echocardiogram. The echocardiogram demonstrated a dilated right ventricle (Video 1), a D-shaped septum (Video 2), and McConnell’s sign (Video 3).

**Video 1:** Parasternal long axis (PLAX) view showing a dilated right ventricle (RV)

**Video 2:** Parasternal short axis (PSAX) view showing a D-shaped septum

**Video 3:** Apical four-chambers view showing McConnell’s sign

**Figure 2:**
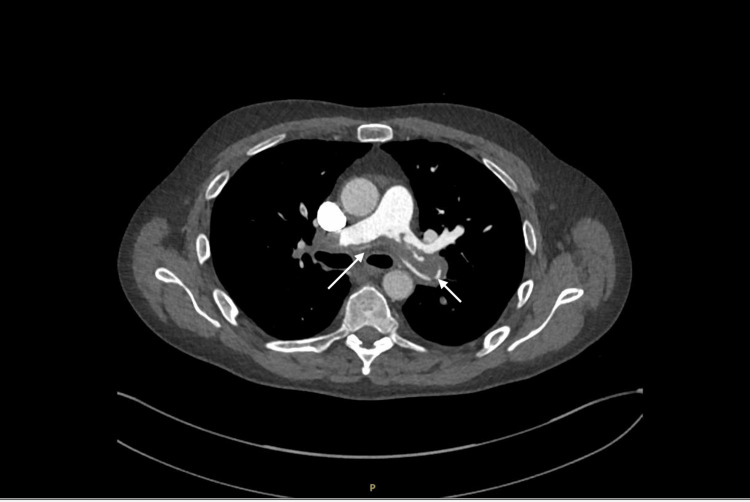
CTPA showing a saddle-shaped embolus (white arrows) CTPA: Computed tomography pulmonary angiography

Subsequently, the blood test results were obtained and were as follows: Troponin I: 28.3 ng/L (reference range: < 2.5 ng/L), N-terminal pro-B-type natriuretic peptide (NT-pro BNP; reference range: < 400 ng/L): 13,151 ng/L, and D-dimer: >20 µg/ml (reference range: < 0.5 ug/ml). Chest X-ray shows no abnormalities and computed tomography pulmonary angiography (CTPA) showed a saddle-shaped thrombus in the pulmonary trunk, extending into the main pulmonary arteries and distal branches (Figure 2).

Differential diagnosis

STEMI was initially suspected but was ruled out based on echocardiographic findings, which showed normal regional wall motion and no features suggestive of ischemia. Ascending aortic dissection with extension to the right coronary artery was excluded due to the absence of imaging evidence on CT angiography and normal aortic morphology. Prinzmetal angina was considered but ruled out as there was no transient ST-segment elevation on serial ECGs. Brugada syndrome was excluded as the ECG did not display the characteristic coved-type ST-segment elevation in leads V1-V3. Acute pericarditis was ruled out due to the absence of diffuse ST-segment elevation or PR depression on ECG and no pericardial effusion on echocardiography.

Treatment and outcome

The patient was treated with anticoagulation, initially with dalteparin, and later transitioned to apixaban. Mechanical thrombectomy was chosen over thrombolysis because the patient was hemodynamically stable at presentation and the multidisciplinary team (MDT) meeting reviewed the imaging, identified both acute and chronic PE (consistent with the patient’s past medical history of PE), and determined that mechanical thrombectomy was the most appropriate initial intervention. During the procedure, only small, white, rubbery organized clots (indicative of chronic thrombi) were retrieved despite repeated aspiration passes. Post-aspiration pulmonary artery pressures remained essentially unchanged, and the follow-up pulmonary angiogram showed no significant improvement. The patient was referred to the Pulmonary Vascular Disease Unit (PVDU) for further evaluation. At the PVDU, the patient was assessed and found to be operable for pulmonary endarterectomy due to the significant thromboembolic burden, the relatively proximal location of the disease, and the presence of moderate pulmonary hypertension (mean pulmonary artery pressure (mPAP) 34 mmHg and PVR 445.6 dyn·s·cm⁻⁵). An MDT at the PVDU concluded that surgery was technically feasible. Treatment with tadalafil 40 mg once daily was initiated to manage pulmonary hypertension and improve fluid status, and diuretics were adjusted by reducing furosemide to 20 mg OD and spironolactone to 25 mg OD to address his low potassium levels (3.2 mmol/L). Despite initial stabilization and mechanical thrombectomy, the patient succumbed to complications before the scheduled surgery.

## Discussion

Differentiating between PE and STEMI is challenging due to overlapping clinical and electrocardiographic features. PE typically presents with sinus tachycardia or non-specific ST-T wave changes. However, ST-segment elevation is an uncommon finding, often linked to acute RV strain, ischemia, or left ventricular underfilling caused by massive PE [1,5]. In this case, the patient’s history of prior thromboembolism and atypical presentation of anterior ST elevation prompted further investigation using bedside echocardiography. The detection of McConnell’s sign, a finding with high specificity (94%) for PE, was pivotal. This highlights the utility of echocardiography in cases where ECG findings are inconclusive or misleading. However, McConnell’s sign has its limitations, such as lower sensitivity, necessitating further diagnostic confirmation. Other echocardiographic features, such as tricuspid regurgitation or RV hypokinesis, may indicate massive PE and should be considered [2,6]. The elevated troponin and NT-proBNP levels in this patient are indicative of RV strain, commonly seen in massive PE, and carry significant prognostic value. Elevated troponin levels have been associated with increased mortality and adverse outcomes in patients with acute PE. According to the European Society of Cardiology (ESC) guidelines on PE, elevated troponin is a marker of myocardial injury and should prompt risk stratification in combination with clinical and imaging findings. Similarly, NT-proBNP levels >600 pg/mL have been linked to increased short-term mortality in PE patients [5]. These biomarkers, used alongside imaging findings such as RV dilation and interventricular septal flattening on echocardiography, can help identify high-risk patients requiring urgent intervention [5,7,8]. A meta-analysis of 19 studies found that clinical impression alone had a sensitivity of 85% and specificity of 51% for PE diagnosis. This underscores the limitations of relying solely on clinical evaluation and emphasizes the importance of multimodal diagnostic approaches, combining imaging, biomarkers, and clinical findings for accurate diagnosis and risk stratification [9]. CT pulmonary angiography remains the gold standard for diagnosing PE, offering high sensitivity and specificity, along with advantages such as rapid diagnosis and visualization of thrombus burden. However, it has limitations, including the risks associated with radiation exposure and contrast nephropathy. In scenarios where CT is contraindicated, ventilation-perfusion (V/Q) scanning serves as an alternative diagnostic modality, though its role in massive PE requires further elaboration [10,11]. Despite mechanical thrombectomy, the patient succumbed to complications. This highlights the critical nature of early recognition and aggressive management in massive PE. Surgical interventions, such as pulmonary endarterectomy, remain the definitive treatment for technically operable chronic thromboembolic pulmonary hypertension but are limited by timing and patient stability [12,13].

## Conclusions

This case highlights the importance of integrating clinical history, ECG findings, biomarkers, and imaging to achieve an accurate diagnosis of PE, especially in atypical presentations. While the ECG findings did not align with typical patterns of STEMI, a high index of suspicion and early use of bedside echocardiography, supported by advanced imaging, were crucial in promptly identifying the underlying pathology. Multidisciplinary collaboration is essential in managing complex cases of PE, ensuring timely and effective intervention. To improve outcomes in similar scenarios, incorporating PE-specific protocols, such as the use of risk stratification tools like the PESI score or sPESI, and algorithms for thrombolysis in massive PE, can guide clinical decision-making. This case underscores the importance of continued education for clinicians on recognizing atypical presentations of PE, as well as the potential for research to refine diagnostic and management pathways for this life-threatening condition.
